# Computer-aided diagnosis of breast microcalcifications based on dual-tree complex wavelet transform

**DOI:** 10.1186/1475-925X-11-96

**Published:** 2012-12-19

**Authors:** Wushuai Jian, Xueyan Sun, Shuqian Luo

**Affiliations:** 1 College of Biomedical Engineering, Capital Medical University, Beijing, 100069, People's Republic of China

**Keywords:** Micro-calcifications, Computer-aided diagnosis, Dual-tree complex wavelet transform

## Abstract

**Background:**

Digital mammography is the most reliable imaging modality for breast carcinoma diagnosis and breast micro-calcifications is regarded as one of the most important signs on imaging diagnosis. In this paper, a computer-aided diagnosis (CAD) system is presented for breast micro-calcifications based on dual-tree complex wavelet transform (DT-CWT) to facilitate radiologists like double reading.

**Methods:**

Firstly, 25 abnormal ROIs were extracted according to the center and diameter of the lesions manually and 25 normal ROIs were selected randomly. Then micro-calcifications were segmented by combining space and frequency domain techniques. We extracted three texture features based on wavelet (Haar, DB4, DT-CWT) transform. Totally 14 descriptors were introduced to define the characteristics of the suspicious micro-calcifications. Principal Component Analysis (PCA) was used to transform these descriptors to a compact and efficient vector expression. Support Vector Machine (SVM) classifier was used to classify potential micro-calcifications. Finally, we used the receiver operating characteristic (ROC) curve and free-response operating characteristic (FROC) curve to evaluate the performance of the CAD system.

**Results:**

The results of SVM classifications based on different wavelets shows DT-CWT has a better performance. Compared with other results, DT-CWT method achieved an accuracy of 96% and 100% for the classification of normal and abnormal ROIs, and the classification of benign and malignant micro-calcifications respectively. In FROC analysis, our CAD system for clinical dataset detection achieved a sensitivity of 83.5% at a false positive per image of 1.85.

**Conclusions:**

Compared with general wavelets, DT-CWT could describe the features more effectively, and our CAD system had a competitive performance.

## Background

Breast cancer is one of the most common cancers among female diseases all over the world. As the causes are unknown, early diagnosis and treatment is particularly important in reducing the mortality rate. Currently, the most effective method for early detection of breast cancer is mammography [1,2], and micro-calcifications are important sign of early-stage breast cancer, which are tiny deposits of calcium that appear on the mammograms as bright spots. Because of subtle findings, radiologist distraction or complex architecture the number of false negative mammograms are higher during evaluating screening mammograms. Consequently, about 10-30% cases are missed during the routine check [1]. With the development of computer technology, a computer-aided diagnosis system comes into being, which can be useful for the radiologists as a second reader. Balleyguier et al. [3] showed that the use of CAD is more useful for the junior radiologist with an improvement in sensitivity from 61.9% to 84.6% compared to a slight improvement from 76.9% to 84.6% for the experienced radiologist.

Generally, a typical CAD system consists of four stages including image preprocessing, extraction of ROIs, detection of micro-calcifications, feature extraction and classification.

### Detection of micro-calcifications

There are many methods of micro-calcifications detection in mammograms, mainly including traditional image processing methods, filtering, threshold algorithm, neural network, SVM, etc. Boccignone G et al. [4] proposed a method combining multi-resolution analysis based on wavelet transform and threshold segmentation based on Renyi entropy. Nakayama R et al. [5] decomposed the mammograms using orthogonal 2-D wavelet transform to obtain Hessian matrix of every pixel, and then micro-calcifications were detected by computing the Eigen values of matrixes. In [6] the breast area was first segmented using morphological filtering and threshold method, then the difference image was obtained by subtracting the noise-suppressed image from the enhanced image of the breast area. Finally, micro-calcifications were segmented by classification of difference images based on the neural network classifier. Melloul M et al. [7] described a threshold segmentation algorithm using entropy to select the threshold automatically and freely. All algorithms mentioned above had their advantages and drawbacks respectively.

### Feature extraction and classification

According to Breast Imaging Reporting and Data System of the American Radiology College [8], the characterizations of micro-calcifications include size, shape and distribution. Currently, the descriptions of the extraction area are mainly gray features, shape features and texture features. Gray features reflect the density of breast tissue and contrast between the lesion and surrounding tissue. The common used features are mean, variance, contrast, etc. Kinoshita SK et al. [9] calculated the histogram statistical characters of breast area, including mean, variance, skewness, kurtosis and entropy. Reference [9] used particle size character to describe the structures distribution of the different size, and the Leyden domain characterization to describe the distribution of linear structure. Yu S et al. [10] used mixing features consisting of wavelet features and gray level statistical features as inputs to a multilayer neural network. The feature space describing the mammograms is often large and complex. Therefore, feature selection is an essential work. The common methods include principal component analysis, linear decision analysis, logistic regression, backward selection, one-dimensional analysis and genetic algorithm. There are also many classification methods, including linear discriminate analysis, artificial neural network, Bayesian methods, rule-based detection methods, decision tree, etc. [11]. Besides, many studies used the mixed classification [12].

### DT-CWT

DT-CWT, proposed by Kingsbury [13] is a recent enhancement to the Discrete Wavelet Transform (DWT). Compared to DWT, it is nearly shift-invariant, good multi-direction analysis and limited data redundancy which make it appropriate for feature extraction. High directional selectivity is useful for texture analysis.

**Figure 1:**
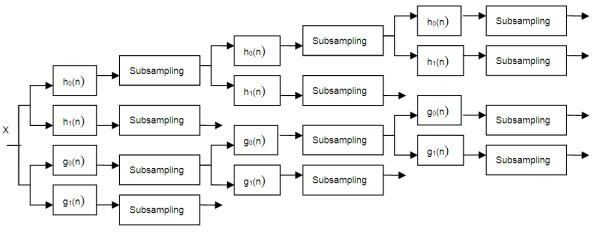
**Practical implementation of DT-CWT on 1-D signal.** X stands for the input signal. h0 (n) and h1 (n) represent the low-pass and high-pass filter pair for the upper filter bank, g_0_ (n) and g_1_ (n) represent the low-pass and high-pass filter pair for the lower filter bank, respectively.

DT-CWT employs two real DWTs; the first gives the real part of the transform while the second gives the imaginary part [14]. Figure 1 shows the framework scheme of DT-CWT for 1-D signal. In the figure, *X* stands for the input signal.*h*_0_ (*n*) and *h*_1_ (*n*) represent the low-pass and high-pass filter pair for the upper filter bank, *g*_0_ (*n*) and *g*_1_ (*n*) represents the low-pass and high-pass filter pair for the lower filter bank, respectively. 2-D DT-CWT is the extension of 1-D DT-CWT. It is essentially performs 1-D DT-CWT on rows and columns of the image in parallel. After 2-D DT-CWT, consisting of four parallel 2-D DWT, we can get six directional sub-bands (±15^0^, ±45^0^, ±75^0^) and the first and latter two parallel transforms produce the real and imaginary part of the six sub-bands, respectively.

The idea behind DT-CWT is inspired from Fourier Transform (FT) which does not suffer from shift variance. This property of FT is based on complex-valued oscillating signals which form a Hilbert pair out of phase components.

Kingsbury proposed to construct a complex-valued wavelet basis which also forms a Hilbert pair.

(1) $$\psi_{c}\left(x\right)=\psi_{r}\left(x\right)+j\psi_{i}\left(x\right)$$

Therefore, DT-CWT can decompose a signal into real and imaginary components as FT. these components are obtained separately using different filter banks. The proposed wavelets in [15] were used in this paper.

## Methods and results

### Dataset and ROI selection

**Figure 2:**
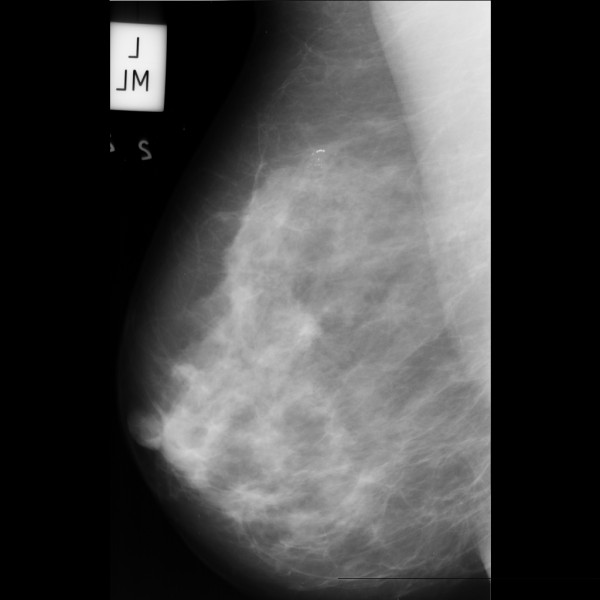
**Typical mammogram in MLO view from MIAS.** The mammograms show micro-calcifications in the upper quadrant of the left breast.

**Figure 3:**
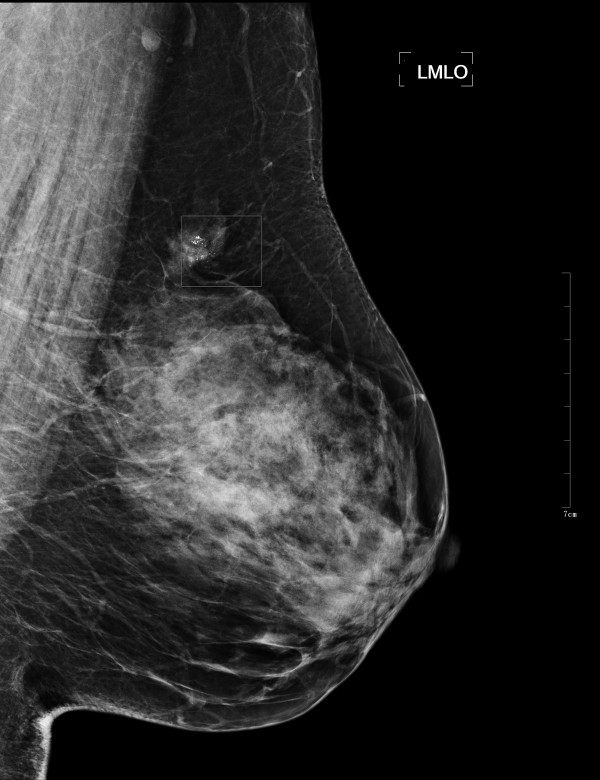
**Typical mammogram in MLO view from clinical dataset.** In the ROI demonstrated there are micro-calcifications in the upper quadrant of the left breast.

The mammograms used in our experiments were selected from the Mammography Image Analysis Society (MIAS) and a clinical dataset of mammograms from the Capital Medical University affiliated hospital. The study was approved by the Ethics Committees of Capital Medical University. ROIs of clinical dataset of mammograms selected and decided by four experienced senior radiologists. MIAS is open access to researchers. This dataset contains 322 images, in which there are 207 normal cases and 115 abnormal cases. Each mammogram is of 1024×1024 pixels, with a spatial resolution of 50 μm/pixel. Figure 2 and Figure 3 show a typical mammogram in MLO view from MIAS and clinical dataset respectively. The coordinates of abnormality center and the approximate radius are provided. Knowing the location and the approximate size of abnormality allows us to manually extract ROIs with proper dimension.

**Figure 4:**
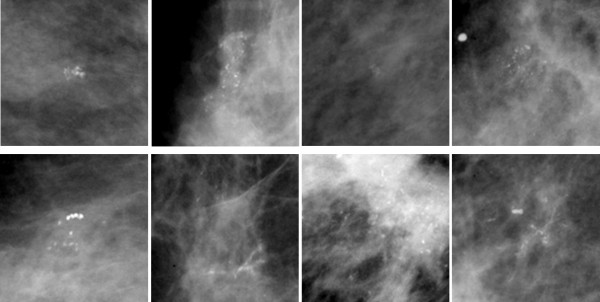
**ROIs containing micro-calcifications.** Totally eight ROIs were selected, and every ROI included a number of micro-calcifications with varied shape, size and distribution.

In our experiment, we used 50 ROIs with a size of 128×128 pixels, in which there are 25 normal ROIs and 25 ROIs containing micro-calcifications. Figure 4 shows eight ROIs containing micro-calcifications. After we obtained the ROIs, we stretched their gray scales to [0, 255] according to the following expression:

(2) $$G_{1}\left(x,y\right)=\left(G_{0}\left(x,y\right)-\min\left(G_{0}\right)\right)\times \frac{255}{\max\left(G_{0}\right)-\min\left(G_{0}\right)}$$

Where *G*_0_(*x, y*) is the original grey value of the point(x, y), *G*_1_(*x, y*) is the grey value of the point(x, y) in the ROI after grey normalization.

### Detection of micro-calcifications

In the mammograms, micro-calcifications are usually brighter and smaller than the surrounding normal tissue. From the points of frequency domain, the micro-calcifications mainly consist of high-frequency component, while the background mainly consists of the low frequency component. In this section, we used a method combining difference image technique and wavelet transform. The steps are as follows:

1. Difference image technique

Since the Laplace filtering can sharp a figure and enhance the high-frequency components, while smooth filtering can blur a figure and suppress the high-frequency components, the high-frequency image are constructed by the following scheme. f_1_ represents figure filtered by Laplace operator, f_2_ represents figure filtered by 3×3 smooth filtering operator f_3_ was obtained Subtract f_2_ from f_1_ and image. Then detect the edge of f_3_ using Kirsch operator and do binary segmentation.

2. Wavelet transform

By the simulation and analysis, we think the high-pass sub-band of first level consists of high-frequency components and the high-pass sub-band of fourth level consists of some low-frequency components. Thus, the original image was decomposed by Daubechies wavelet at level four and the coefficients of the first layer and the fourth layer were set to zero. Then the wavelet reconstruction was done. Finally threshold segmentation was done.

**Figure 5:**
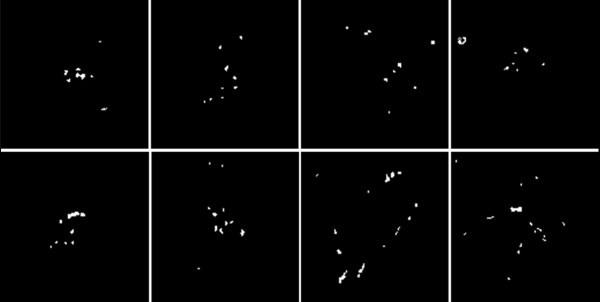
**Results of micro-calcifications detection.** Micro-calcifications corresponding to Figure 3 were detected effectively.

3. After the first step, we got a binary image containing micro-calcifications and noise. Also, after the second step, we got a binary image containing micro-calcifications and background. Then, logical ‘And’ operation was taken on the results of the previous two steps. The partial results were shown in Figure 5.

### Feature extraction

Feature extraction and selection are important steps in CAD. DT-CWT has a better directional selection than general DWT, which describes the directional features of texture preferably. So DT-CWT was used to get the wavelet features.

In order to reduce the calculation burden and complexity, we used PCA to choose a few key features from the potential micro-calcifications features. PCA can reduce the high dimensional correlated features into low dimensional features. In PCA, the symmetric covariance matrix or symmetric correlation matrix are calculated, and the eigenvalues and eigenvectors of these matrix are calculated. By PCA, a few irrelevant principal components are removed so that can retain the original information as complete as possible.

**Figure 6:**
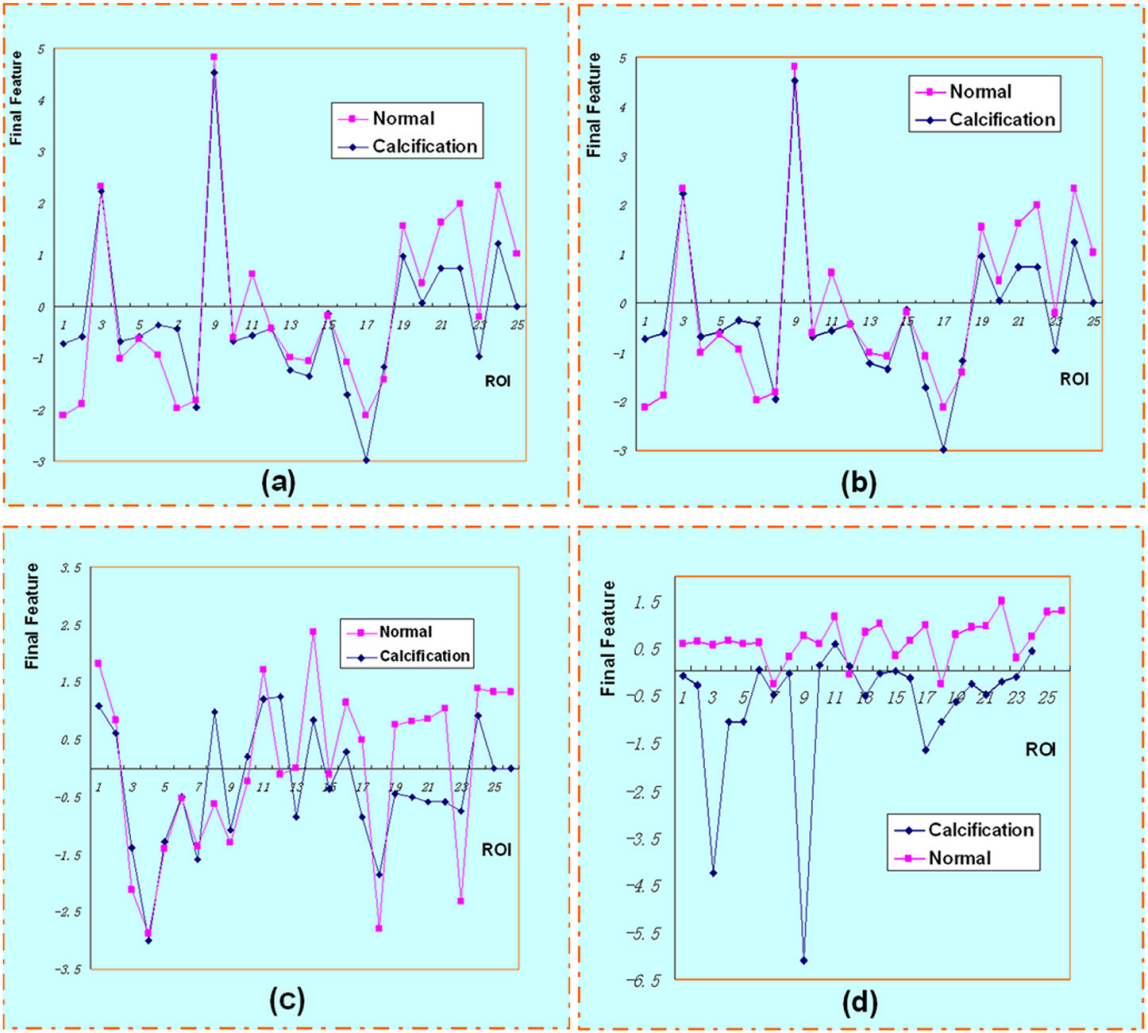
**Final features of 50 ROIs. a**. without wavelet **b**. Haar **c**. DB4 **d**. DT-CWT ROIs of the two categories have differences and DT-CWT had a better performance.

In our work, we choose fourteen features, including 2-nd moment, 4-th moment, 9 texture features and 3 wavelet coefficients. We used different wavelets in extracting the features. Then we transformed the 14 features to 4 linearly independent features. We have gotten a final feature called comprehensive score according to the contributions of the former 4 features. The final features of 50 ROIs are shown in Figure 6. The figures shows ROIs of the two categories have differences, and DT-CWT indeed has a better performance. The detailed descriptions of features used were as follows:

1. If the gray scale, gray average and histogram of an image are *K*, *μ* and *h* (*κ*), *κ*=0, 1, 2, …, *K*-1 respectively, the n-order moment of the mean of histogram is defined as:

(3) $$m_{n}=\frac{1}{N}\sum_{k=0}^{K-1}{\left(k-\mu \right)}^{n}h\left(k\right)$$

The second-order moment, also known as variance, describes the visual roughness of the image. The third-order and fourth-order moment are called inclination and kurtosis, respectively, reflecting the asymmetry and the uniformity of the histogram. This paper used the second-order and fourth-order moment.

2. Texture features were calculated using the gray level co-occurrence matrixes (GLCM) [16]. The results are 9 texture parameters. Suppose GLCM are defined as *Cij*. The angular second moment, inertia, inverse difference moment, entropy, correlation, sum average, difference average, sum entropy and difference entropy are computed as follows:

(4) $$\begin{array}{l} {\text{T}}_{1}=\sum_{i=0}^{K-1}\sum_{j=0}^{K-1}{\text{C}}_{\mathrm{ij}}^{2}\\ T_{2}=\sum_{i=0}^{K-1}\sum_{j=0}^{K-1}{\left(i-j\right)}^{2}C_{\mathrm{ij}}\\ T_{3}=\sum_{i=0}^{K-1}\sum_{j=0}^{K-1}\frac{1}{1+{\left(i-j\right)}^{2}}C_{\mathrm{ij}}\\ T_{4}=-\sum_{i=0}^{K-1}\sum_{j=0}^{K-1}C_{\mathrm{ij}}\log C_{\mathrm{ij}}\\ T_{5}=\frac{\sum_{i=0}^{K-1}\sum_{j=0}^{K-1}\left(\mathrm{ij}\right)C_{\mathrm{ij}}-\mu_{x}\mu_{y}}{\sigma_{x}\sigma_{y}}\\ T_{6}=\sum_{k=0}^{2K-2}kC_{x+y}\left(k\right)\\ T_{7}=\sum_{k=0}^{K-1}kC_{x-y}(k)\\ T_{8}=-\sum_{k=0}^{2K-2}C_{x+y}\left(k\right)\log\left\{C_{x+y}\left(k\right)\right\}\\ T_{9}=-\sum_{k=0}^{K-1}C_{x-y}\left(k\right)\log\left\{C_{x-y}\left(k\right)\right\} \end{array}$$

3. We directly decomposed the ROIs using 2-D DT-CWT and 12 sub-bands to get the wavelet coefficients. Then use the wavelet coefficients as the characteristics to classify the lesions.

4. The area is estimated according to the size of the image foreground. The area is measured by the number of pixels roughly. The different weights are added to the different pixels to compensate for the discrete pixels description of the continuous image. The Euler number (E) is a topology descriptor of a region, which describes the connectivity of the region. For a given area, the number of holes (H) and the number of connectivity crew(C) in the region are commonly used in the topological properties. We get E = C-H. In our paper we computed the area and Euler number of the micro-calcifications.

### Classification based on SVM

In order to get a quantitative result we used the SVM classifier. SVM was first introduced by Vapnik and it offers several advantages such as better performance in higher dimension space [17]. The basic idea of SVM is to find a hyper-plane which can best separate the input feature vectors of two classes while maximizing the distance from either class to the hyper-plane. In this paper, we use nonlinear SVM with the quadratic and cubic Polynomial Kemel functions.

The classification process included two steps. In the first step, samples corresponding to benign and malignant cases were labeled into a single class named abnormal class, while the samples not containing micro-calcifications were denoted by normal cases. In the second step, the task was to discriminate between benign and malignant samples. These two steps were both a binary classification problem.

In order to have an adequately representative training set, we adopted a leave-one-out cross-validation in the experiments. In the first step, we accomplished fifty different runs, where forty-nine images were used for training and the remaining one for testing. Similarly, in the second step we accomplished twenty-four different runs, where twenty-three images were used for training and the rest one for testing. The correct recognition rates (accuracy) of different wavelets are shown in Table 1, while the accuracy is defined as the ratio of correctly classified samples versus all samples. Table 2 shows some classification results of other references using different wavelets. Compared with other results, DT-CWT method achieved an accuracy of 96% and 100% for the classification of normal and abnormal and the classification of benign and malignant, respectively. The results also show that the generalizability of this approach is quite well.

**Table 1:** Classification results of different wavelets

**Accuracy**	**Without wavelet**	**Haar**	**DB4**	**DT-CWT**
Classification results of normal and abnormal ROIs	90%	90%	92%	96%
Classification results of benign and malignant micro-calcifications	95.8%	91.7%	87.5%	100%

**Table 2:** Classification results of other methods

				
**Method**	Verma B [18]	Panchal R [19]	Lee S [20]	Ren JC [21]
**Accuracy**	83.3%	100%	95%	98.1%

### Performance evaluation of CAD

**Figure 7:**
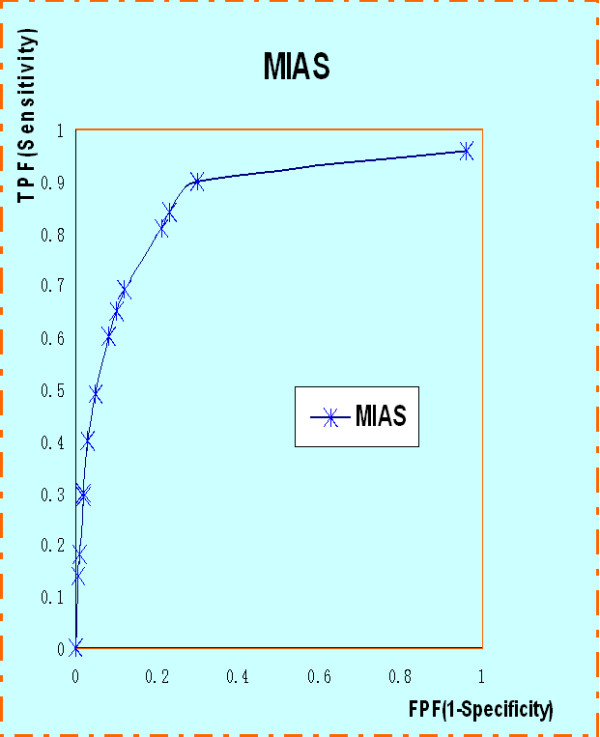
ROC curve of MIAS dataset.

**Figure 8:**
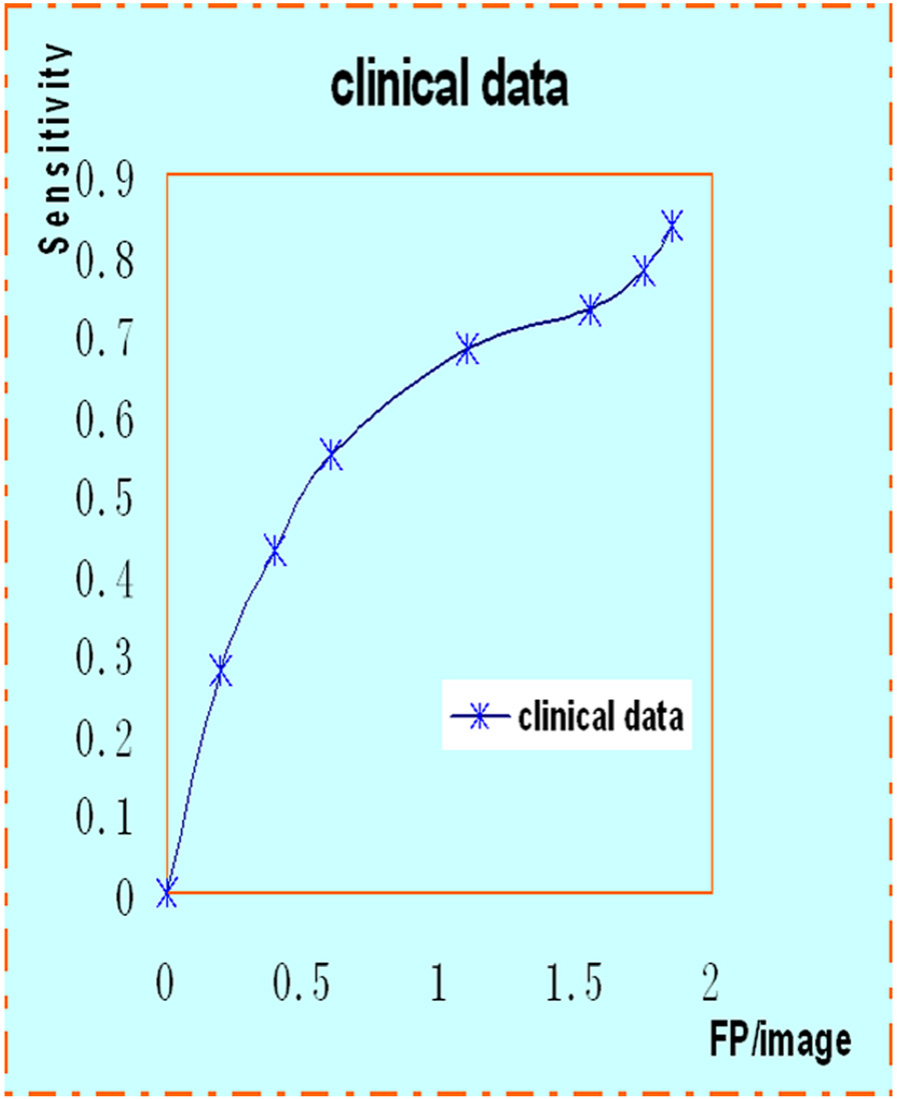
**FROC curve of clinical data.** A sensitivity value of 83% is obtained at a rate of 1.85 FP per image.

The ROC curve and the FROC analysis are common methods in evaluating CAD performance. In the ROC method, the observer classifies each image as normal or abnormal. The ROC curve is defined as a two-dimensional plot of True Positive Fraction (TPF) and False Positive Fraction (FPF). The ROC curve of MIAS is shown in Figure 7. Similarly, the FROC is used to assess the ability to correctly detect the abnormalities. The FROC curve is obtained by plotting the sensitivity, synonymous with TPF, versus the number of false positives per image (FP/image). As shown in the Figure 8, a sensitivity value of 83% is obtained at a rate of 1.85 FP per image.

## Discussion

Computer-aided detection (CADe) systems address the problem that radiologists often miss signs of cancers that are retrospectively visible in mammograms. Furthermore, computer-aided diagnosis (CADx) systems have been proposed that assist the radiologist in the classification of mammographic lesions as benign or malignant [22]. Key CAD techniques developed recently for breast cancer, including detection of calcifications, detection of masses, detection of architectural distortion, detection of bilateral asymmetry, image enhancement, and image retrieval [23]. Deserno TM et al. [24] recently presented an implementation of a SVM-based CBIR (content-based image retrieval) system for CADx in screening mammography.

This paper proposed a new CAD method of micro-calcifications based on DT-CWT. The new diagnosis algorithm to detect and classify micro-calcifications is verified by MIAS mammograms and clinical dataset. By combining special difference image technique and wavelet transform, the suspicious micro-calcifications can be effectively segmented from mammograms. It shows DT-CWT is efficient in describing image features. By PCA, merely four principal components out of fourteen original features are obtained to describe the micro-calcifications. SVM is applied to classify the micro-calcifications. Compared with other results, DT-CWT method achieved an accuracy of 96% and 100% to the classification of normal and abnormal and the classification of benign and malignant, respectively. The experiments showed that our attempt is efficient. To provide more reliable evaluation results on the performance of the proposed scheme, a larger database should be used in future work.

Future the scholars will remain focusing on how to improve the performance of CAD. However, there are two issues that limit the current development of CAD. These are the inability to optimize a scheme for clinical impact—current methods only optimize CAD in the absence of a radiologist—and the lack of a figure of merit that quantifies the performance efficiency of CAD [25]. Such a figure of merit could be used to determine how much better performance CAD could obtain and which component of the several techniques employed is the weakest. The future work should be focused on how to solve these issues which may need more efforts. Furthermore, CAD must not be responsible for omitting the step of the complete evaluation of mammograms by the radiologist. A CAD system cannot and should not replace the radiologist as either or final interpretation [26].

## Conclusions

In conclusion,compared with other wavelets (Haar, DB4), DT-CWT could describe the features more effectively. The paper showed our CAD system had a competitive performance. The use of a CAD system helps the radiologist as a second reviewer to evaluate screening mammograms.

## Abbreviations

CAD: Computer-aided diagnosis; DT-CWT: Dual-tree complex wavelets transform; ROI: Regions of interest; PCA: Principal component analysis; SVM: Support vector machine; ROC: Receiver operating characteristic; FROC: Free-response operating characteristic.

## Competing interests

The authors declare that they have no competing interests.

## Authors' contributions

WJ conceived of the study, and participated in its design and coordination and helped to draft the manuscript. XS worked on the algorithm design and implementation.SL contributed to discussion and suggestions throughout this topic, including the manuscript writing. All authors read and approved the final manuscript.

## Authors' information

About the Author—Wushuai Jian Currently he is a Ph.D candidate at the College of Biomedical Engineering, Capital Medical University, Beijing, P.R. China. His research interests are medical image processing and phase-contrast imaging.

About the Author—Xueyan Sun received the Master of Science degree from the College of Biomedical Engineering, Capital Medical University, Beijing, P.R. China in 2012. Her research interests include: medical image processing, pattern recognition.

About the Author—Shuqian Luo Currently, he is a Full Professor, College of Biomedical Engineering, Capital Medical University, Beijing, China, director of Medical Imaging Lab, IEEE Senior Member, project Leader: Multi-Modality Medical Image Registration, Brain Tissue Segmentation and Classification, 3D Digitalized Human Brain Atlas, Chinese Digital Human. He is editor of many Chinese journal and principle investigator of many projects, including the National High Technique Research and Development Plan (863 Plan), Project of National Natural Science Foundation. He won Henan Science and Technology Progress Second Place Award, Project of Multi-Functional ECG Analyzer, 1993, and Asian Ten major CT (Computed Tomography) Science Award, Project of Multi-Modality Medical Image Registration, 1999, and Beijing Traditional Chinese Medicine First Place Award, Project of Meridian Adjustment Diagnosis and Therapy System, 2001.3. Prof. Luo has published 150 papers and 6 books.
